# Supplementary material for: The Influence of Carbon Nature on the Catalytic Performance of Ru/C in Levulinic Acid Hydrogenation with Internal Hydrogen Source
Source: Molecules. 2020 Nov 17;25(22):5362. doi: 10.3390/molecules25225362 (PMC7698119; doi:10.3390/molecules25225362)
Supplement: Supplementary file 1 [file molecules-25-05362-s001.pdf]

Supporting Information

The influence of carbon nature on the catalytic performance of Ru/C in levulinic acid hydrogenation with internal hydrogen source

Marcin Jędrzejczyk [1,*], Emilia Soszka [1], Joanna Goscianska [2], Marcin Kozanecki [3], Jacek Grams [1] and Agnieszka M. Ruppert [1,*]

[1] Institute of General and Ecological Chemistry, Faculty of Chemistry, Lodz University of Technology, Żeromskiego 116, 90-924 Łódź, Poland
[2] Faculty of Chemistry, Adam Mickiewicz University in Poznań, Uniwersytetu Poznańskiego 8, 61-614 Poznań, Poland
[3] Department of Molecular Physics, Faculty of Chemistry, Lodz University of Technology, Żeromskiego 116, 90-924 Łódź, Poland
* Correspondence: (M.J.) marcin.jedrzejczyk@p.lodz.pl; (A.M.R.) agnieszka.ruppert@p.lodz.pl

1. *Nitrogen adsorption-desorption isotherms of carbon supports*

Figure S1 depicts nitrogen adsorption-desorption isotherms obtained for carbon materials AC1, AC2, AC3, CWZ, AG, and Norit. Most of them have a shape close to that of the reversible type I isotherm according to IUPAC classification, characteristic for microporous solids. The exceptions are carbon samples Norit and AG whose isotherms are similar to type IV assigned to mesoporous molecular sieves. They have hysteresis loops of H4 type, which confirm the contribution of mesopores in the porous structure of the carbon samples studied.

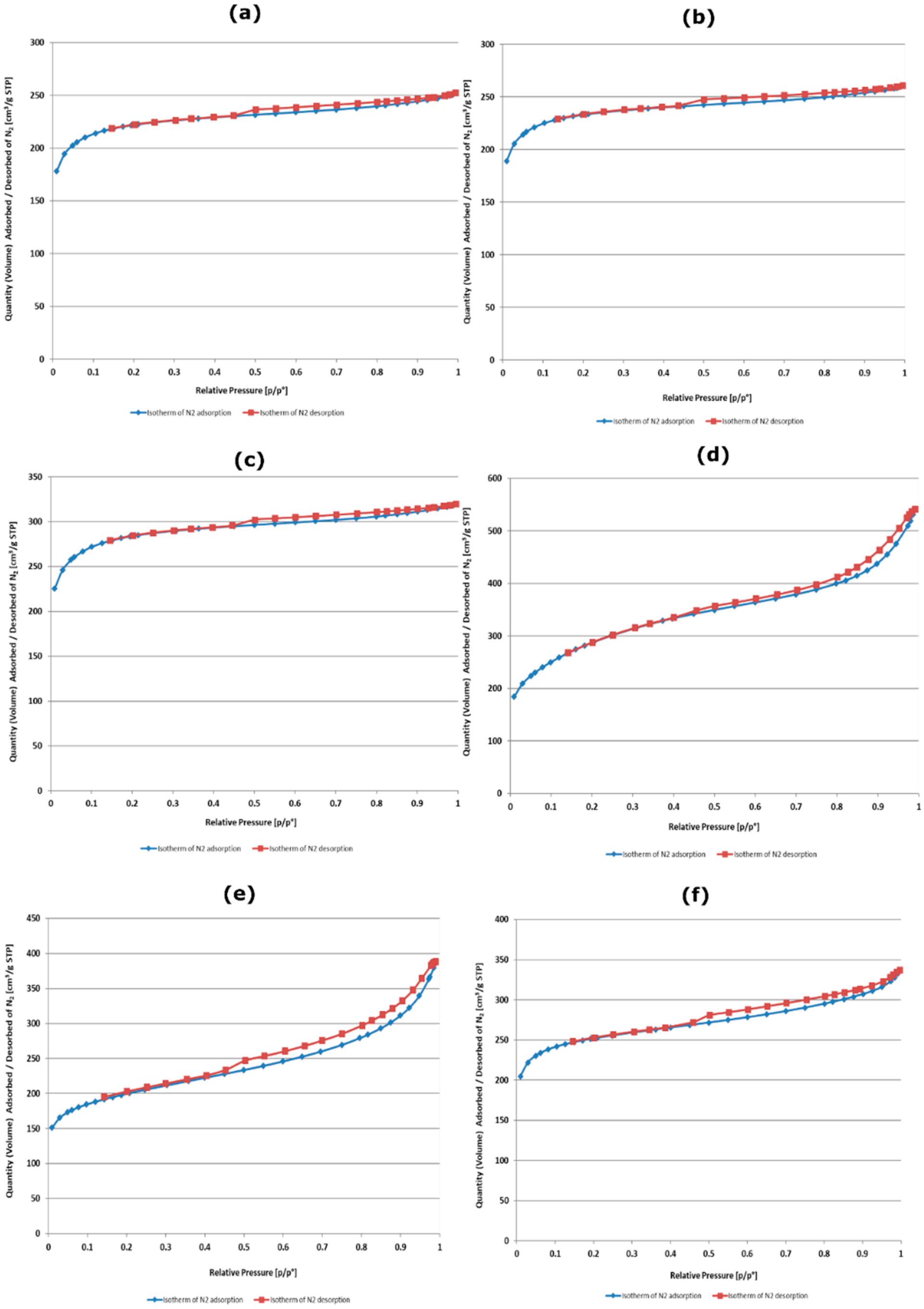

Figure S1. Nitrogen adsorption-desorption isotherms of supports (a) AC1, (b) AC2, (c) AC3, (d) AG, (e) Norit, (f) CWZ.

2. Temperature programmed reduction

The reducibility tests of bare supports are shown in Figure S2. No hydrogen consumption was noted till 450°C. The gentle hydrogen consumption observed in higher temperature is related with the reduction of organic groups present on the carbon surface.

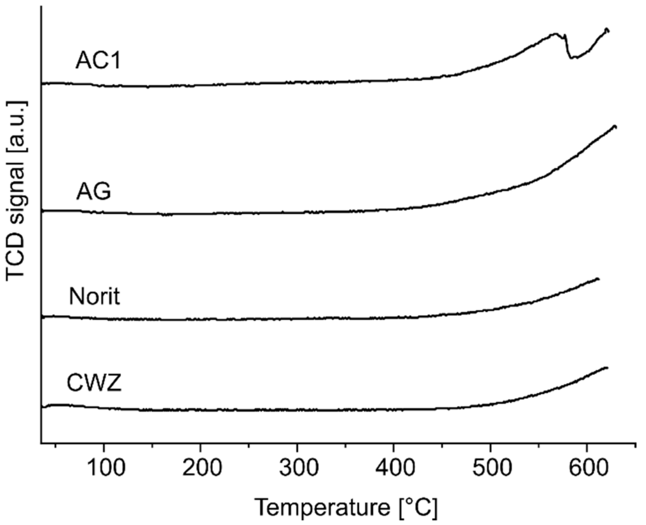

Figure S2. TPR-H_2 profiles of supports.

In order to assess the reduction behavior of ruthenium on the surface of carbon materials, TPR experiment was performed according to the following procedure: reduction of Ru/AC1 catalyst to 400°C, cooling in the atmosphere of hydrogen to room temperature and subsequent reduction to 400°C. The obtained results are presented in Figure S3. The TPR-H_2 profile of the Ru catalyst recorded in the first cycle is discussed in the publication. In the second cycle, the effects of hydrogen uptake were not observed, which suggests a complete reduction of ruthenium and presence of its metallic phase on the AC1 carbon surface after reduction in the first cycle.

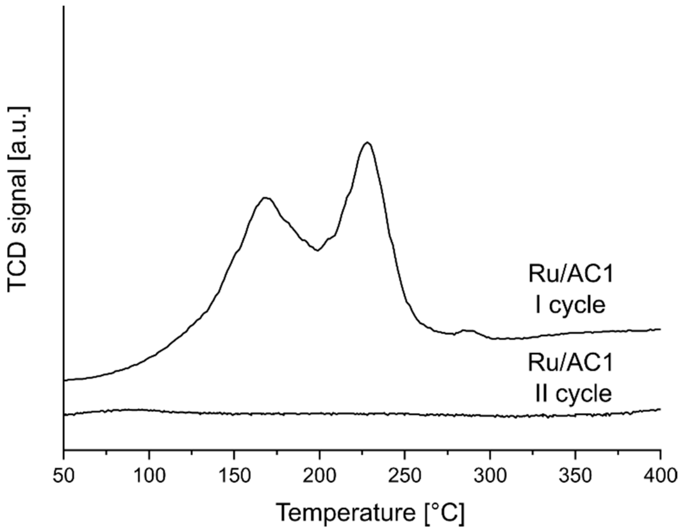

Figure S3. TPR-H_2 profiles of Ru/AC1 catalyst (I and II cycle).

3. Reusability of the catalysts

The stability of the Ru/AC1 catalyst was studied in the LA hydrogenation, the results are shown in Figure S4. The catalyst was recovered after the reaction, washed with water and dried at 100°C for 5 hours and reused. Under these conditions, the Ru/AC1 catalyst showed no change in activity till the fifth reuse cycle that confirms high stability of this material in the reaction conditions.

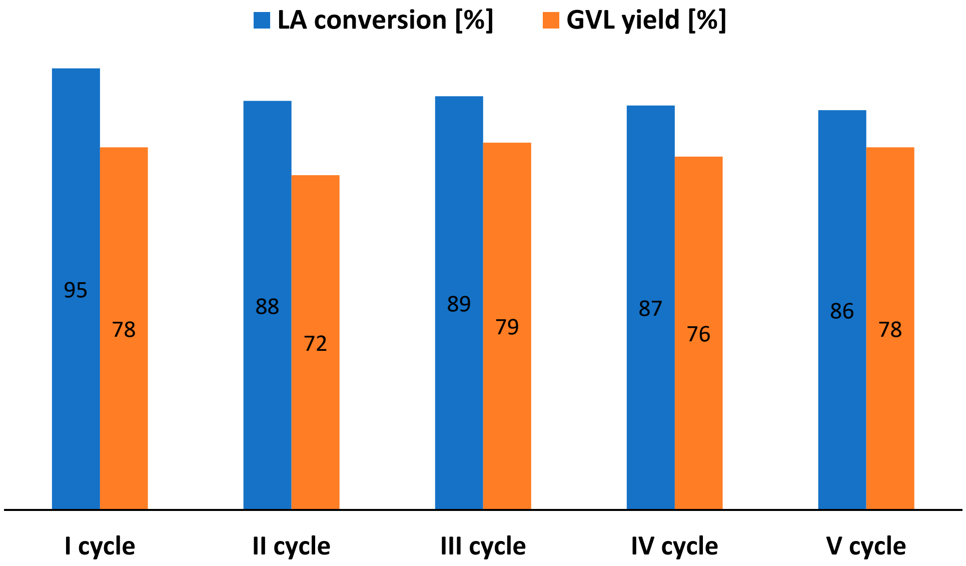

Figure S4. Reusability tests of Ru/AC1 catalyst in LA hydrogenation
